# Superficial Inferior Epigastric Artery (SIEA) Flap For Autologous Breast Reconstruction: A Single-Institution Experience With Technical Pearls

**DOI:** 10.7759/cureus.100806

**Published:** 2026-01-05

**Authors:** Shu Ying Chee, Khairun Izlinda Abdul-Jalil, James Fleming, Konrad Timon, Yen Xian Lee, Colin Morrison

**Affiliations:** 1 Plastic and Reconstructive Surgery, St. Vincent's University Hospital, Dublin, IRL; 2 Plastic and Reconstructive Surgery, University College Dublin School of Medicine, Dublin, IRL

**Keywords:** autologous reconstruction, breast reconstruction, deep inferior epigastric flap, perforator flap, superficial inferior epigastric artery flap

## Abstract

Introduction

Autologous abdominal tissue transfer is considered the gold standard for breast reconstruction, providing natural composition and texture. The superficial inferior epigastric artery (SIEA) flap represents the least invasive option, sparing both the rectus muscle and fascia. However, its adoption remains limited due to concerns regarding arterial complications and donor site morbidity. We aimed to describe our experience with SIEA flaps in carefully selected patients and present technical pearls to optimise outcomes.

Methods

A retrospective chart review was performed of all breast cancer patients undergoing abdominal-based free flap breast reconstruction at a single institution from January 2020 to September 2025. Patients were selected for SIEA reconstruction based on preoperative computed tomography angiography (CTA) demonstrating a dominant superficial system (SIEA diameter ≥1.5 mm) with inadequate deep inferior epigastric artery perforator (DIEP) anatomy. Demographic data, comorbidities, adjuvant therapies, operative details, and complications were recorded. Outcomes were compared descriptively with contemporaneous DIEP flap reconstructions. Categorical variables were analyzed using Fisher's exact test, with statistical significance set at p<0.05. Given the small SIEA cohort size (five flaps in five patients), this study should be considered hypothesis-generating rather than definitive.

Results

During the study period, 170 flaps were performed in 161 patients; 165 DIEP flaps (97%) in 156 patients and five SIEA flaps (3%) in five patients. All SIEA reconstructions were unilateral and immediate. Mean patient age was 48.4 years (SIEA) versus 53.5 years (DIEP). Mean hospital length of stay was 10 days for SIEA patients versus eight days for DIEP patients. At the recipient site, no arterial insufficiency, venous thrombosis, or flap failure occurred in the SIEA cohort. Donor site seroma occurred in 1/5 (20%) SIEA flaps versus 3/165 (1.8%) DIEP flaps (p=0.058). No abdominal dehiscence or hernia occurred in the SIEA group. Overall complication rates were 20% (1/5) for SIEA versus 5.5% (9/165) for DIEP flaps (p=0.264). The limited sample size precludes definitive statistical conclusions.

Conclusion

In highly selected patients with favourable superficial arterial anatomy on preoperative CTA, SIEA flaps can achieve successful breast reconstruction with minimal donor site morbidity. Careful patient selection, meticulous surgical technique incorporating specific technical pearls, and appropriate recipient vessel selection are essential to optimise outcomes. While the trend toward increased seroma formation warrants attention, the complete absence of abdominal wall weakness represents a significant advantage. Larger multi-institutional studies are needed to establish definitive comparative outcomes.

## Introduction

Autologous abdominal tissue transfer is considered the gold standard for breast reconstruction, providing tissue with composition and texture most similar to native breast tissue [1]. The evolution of abdominal-based free tissue transfer has progressed from the transverse rectus abdominis myocutaneous (TRAM) flap to the deep inferior epigastric perforator (DIEP) flap and ultimately to the superficial inferior epigastric artery (SIEA) flap, which was popularised by Grotting in 1991 [1].

The DIEP flap, first described by Koshima and Soeda [2] and popularised by Allen and Treece in the early 1990s [3], has become the preferred choice for abdominal-based breast reconstruction. It relies on one to four perforating vessels from the deep inferior epigastric artery and vein, sparing the rectus abdominis muscle while requiring violation of the anterior rectus sheath [4]. The technique offers sizable flaps with a reliable, lengthy pedicle, but requires technically challenging intramuscular dissection with a steep learning curve [4,5].

The SIEA flap offers theoretical advantages over the DIEP flap by requiring no dissection through fascia or muscle, potentially resulting in reduced donor site morbidity, shorter operative time, and quicker recovery [6,7]. The SIEA typically arises as a direct branch of the common femoral artery 2-5 cm below the inguinal ligament, though it may originate from the profunda femoris or superficial external pudendal artery [8]. The arterial caliber ranges from 1.1 to 1.9 mm [9]. The superficial inferior epigastric vein (SIEV) commonly drains into the femoral vein or great saphenous vein [6].

Despite these theoretical advantages, adoption of the SIEA flap has been limited. Multiple studies have reported higher rates of arterial thrombosis, ranging from 8% to 17%, compared to 1-2% with DIEP flaps [10-12]. Selber et al. reported a 14% total flap loss rate with SIEA flaps versus 0.18% with muscle-sparing TRAM flaps [13], while Coroneos et al. found SIEA flaps had significantly higher rates of re-exploration (20% vs 7%), arterial insufficiency (14% vs 1%), and failure (14% vs 2%) compared to DIEP flaps [10]. Donor site seroma formation has been reported in 20-53% of SIEA reconstructions compared to 2-10% with DIEP flaps [14-16]. These concerns regarding variable anatomy, short pedicle length, increased thrombosis risk, and high seroma rates have led many surgeons to abandon the technique [10,13].

However, recent studies with refined patient selection criteria have demonstrated more favourable outcomes. Henry et al. reported that an SIEA diameter ≥2.0 mm at its origin on preoperative computed tomography angiography (CTA) provides a reliable criterion for flap utilisation [17]. Spiegel and Khan demonstrated that implementing an intra-operative algorithm with strict arterial diameter criteria (≥1.5 mm) eliminated flap failures in their series after initial learning curve [18].

The aim of this study is to describe our institutional experience with SIEA flaps in carefully selected patients based on preoperative imaging criteria, to compare outcomes descriptively with contemporaneous DIEP reconstructions, and to present technical pearls that may optimise success rates and minimise complications.

## Materials and methods

Study design

This retrospective cohort study was conducted in accordance with the Declaration of Helsinki and is reported according to STROBE (Strengthening the Reporting of Observational Studies in Epidemiology) guidelines for cohort studies [19].

Setting and participants

A retrospective chart review was performed of all breast cancer patients undergoing abdominal-based free flap breast reconstruction at St. Vincent's University Hospital, Dublin, Ireland, between January 2020 and September 2025. Patients were excluded if they had undergone previous abdominoplasty, had a history of failed abdominal flap reconstruction, or had insufficient abdominal tissue for reconstruction.

Preoperative planning 

**Figure 1:**
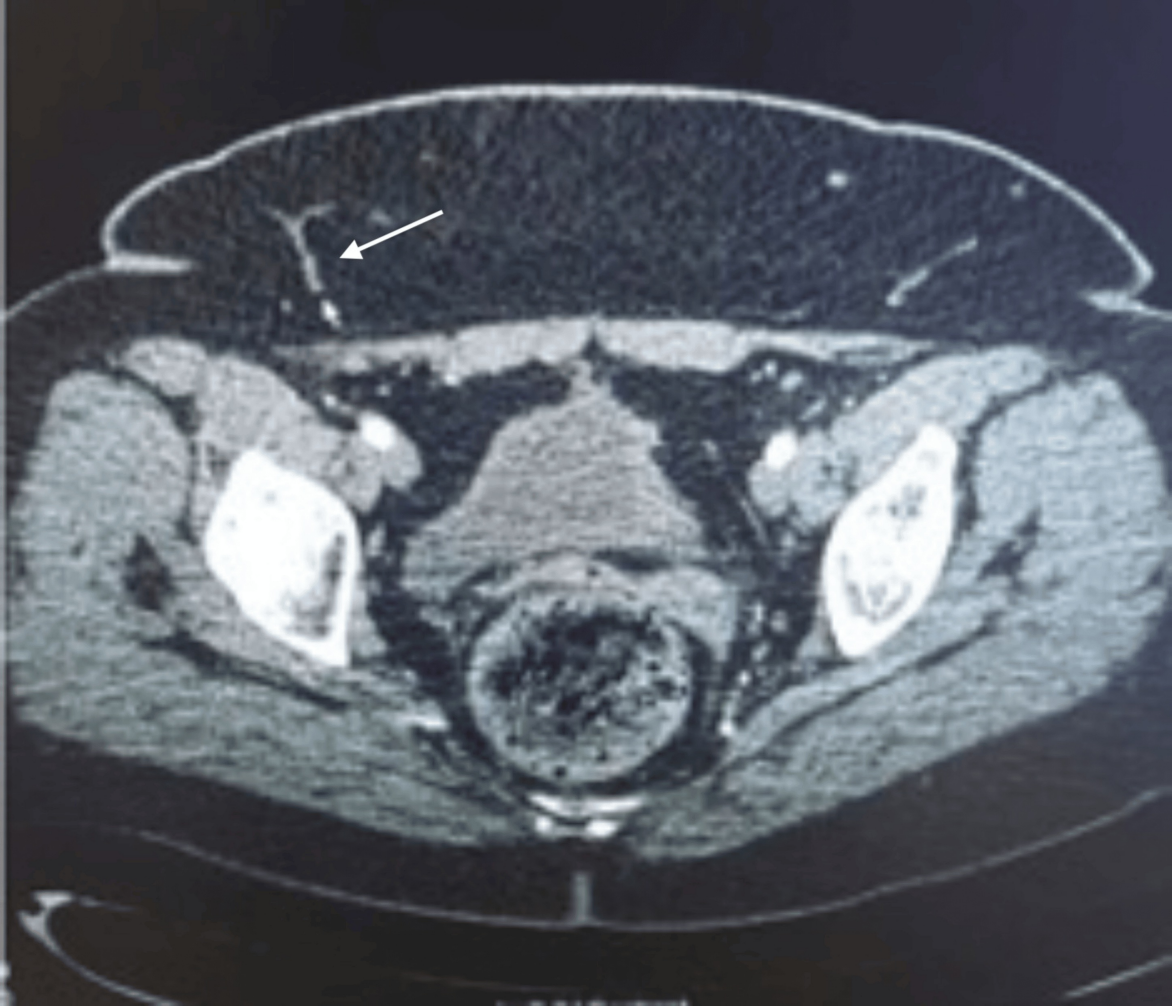
Preoperative CT angiogram (axial view) showing superficial system dominance

**Figure 2:**
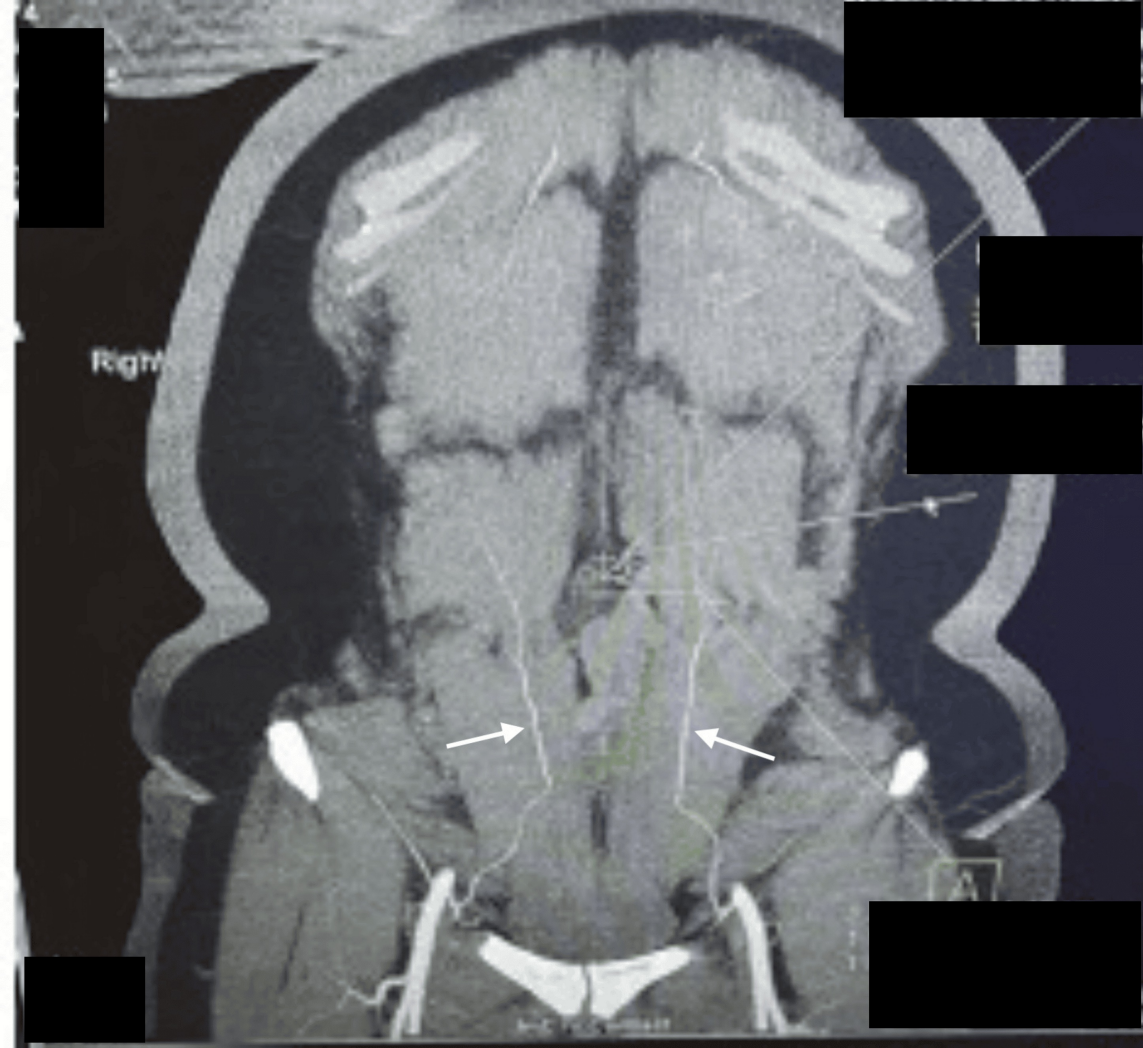
Preoperative CT angiogram (coronal view) showing superficial system dominance

All patients undergoing abdominal-based free tissue breast reconstruction in our unit routinely undergo a preoperative CT angiogram of their vascular anatomy. This allows accurate delineation and selection of the largest-calibre deep inferior epigastric artery (DIEA) vessels, which are preferentially located in the medial row in the peri-umbilical region and typically have a short intramuscular course. All scans are reviewed in conjunction with our radiology colleagues to also assess the superficial vascular system (Figures 1-2).

**Figure 3:**
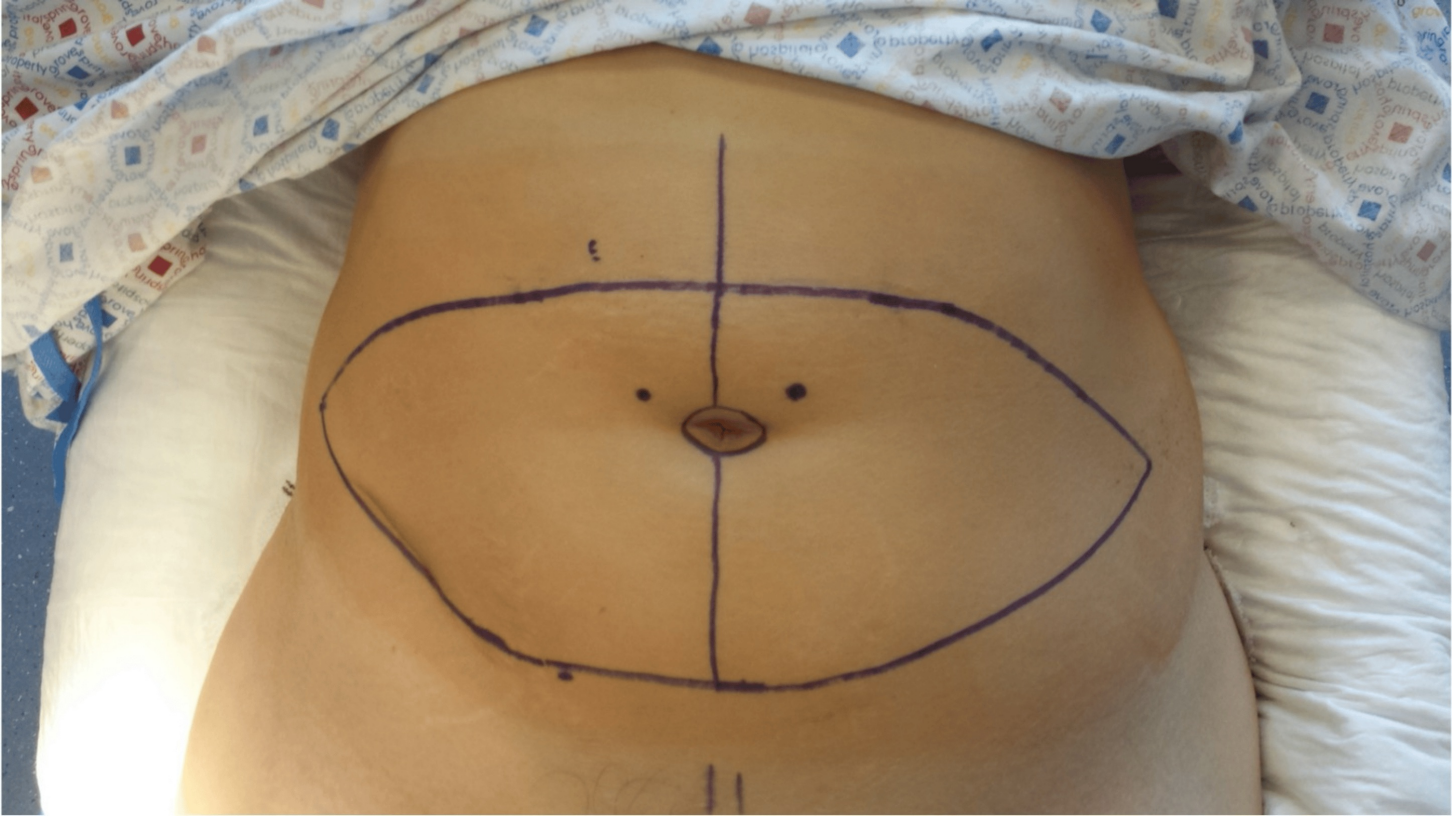
Preoperative flap markings (for both DIEP and SIEA flap) DIEP: deep inferior epigastric perforator; SIEA: superficial inferior epigastric artery

Patients are marked in the supine position using the anterior superior iliac spines (ASIS), umbilicus, and pubic symphysis as anatomical landmarks. Marking is performed according to the standard DIEP flap technique. The peri-umbilical medial row perforators are identified and marked on the skin bilaterally, adjacent to the midline (Figure 3). In addition, a handheld pencil Doppler is used to identify and mark the SIEA, located midway between the ASIS and the pubic symphysis, just superior to the inguinal crease. The SIEV is typically located medially. This approach facilitates planning for either an SIEA or DIEP flap.

The lower abdominal incision is positioned as inferiorly as possible to maximise the likelihood of identifying an SIEA of favourable calibre. The horizontal and vertical dimensions of the flap vary according to individual patient anatomy; however, the vertical height is typically no greater than 12 cm. A pre-existing lower abdominal scar is a relative contraindication to an SIEA flap and may limit inferior flap positioning, as the scar must be incorporated into the flap design.

Preoperatively, an SIEA flap was considered only if both of the following criteria were met: the presence of a clearly dominant SIEA system with an arterial diameter of ≥1.5 mm at its origin, and inadequate or unreliable DIEP perforator anatomy on pre-operative imaging.

**Figure 4:**
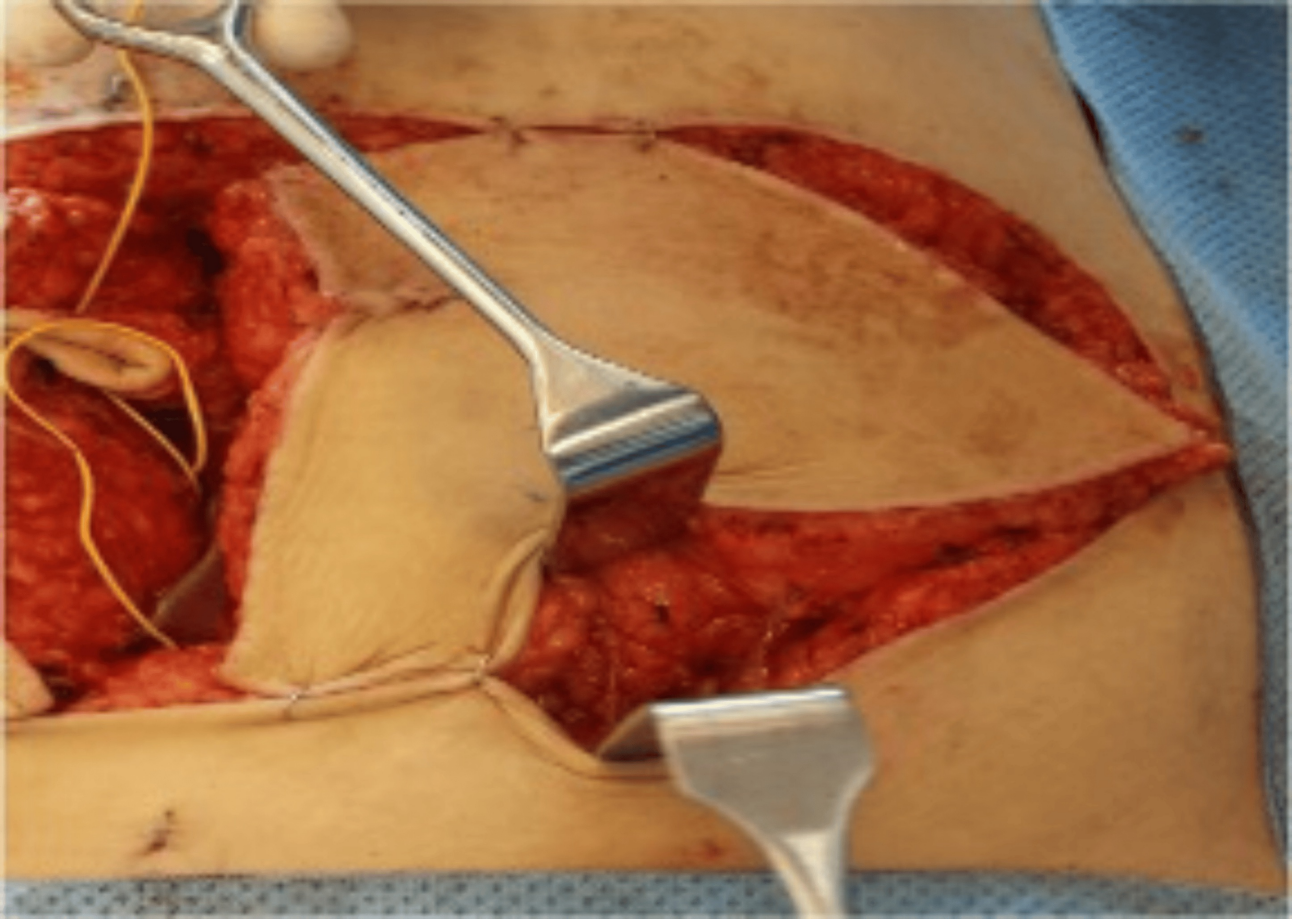
Pedicle identification for SIEA flap SIEA: superficial inferior epigastric artery

**Figure 5:**
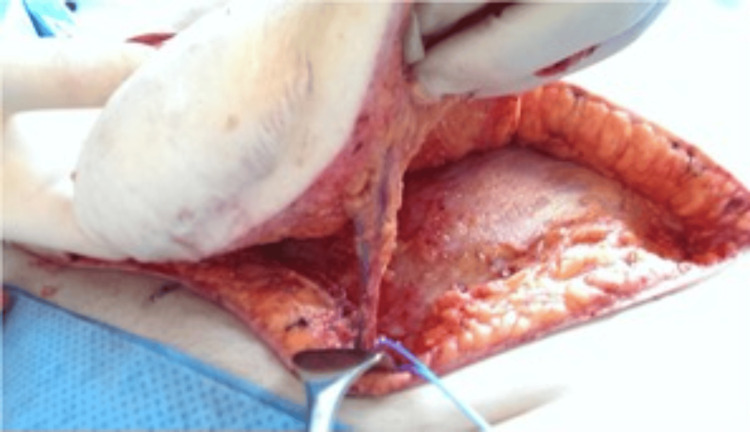
Pedicle dissection for SIEA flap SIEA: superficial inferior epigastric artery

**Figure 6:**
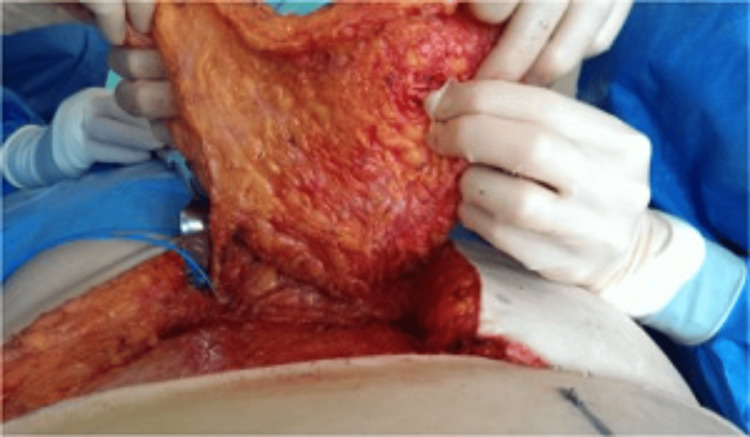
Abdominal wall preservation for SIEA flap SIEA: superficial inferior epigastric artery

Intraoperatively, an SIEA flap was performed if all of the following criteria were met: the SIEA vessels measured at least 2 mm in diameter at the level of the femoral artery or its trunk, the artery demonstrated a palpable pulse with an audible Doppler signal, and the “medial vein” (SIEV) was present and suitable for dissection over a length of at least 6 cm (Figures 4-6).

Variables and data collection

Data were abstracted from electronic medical records using a standardised data collection form. Variables included: (i) Patient characteristics: age, body mass index (BMI), smoking status, diabetes mellitus, reconstruction timing (immediate vs delayed), previous radiation therapy, and previous chemotherapy; (ii) Operative details: flap type (SIEA vs DIEP), recipient vessels (internal mammary artery/vein vs thoracodorsal vessels), number of arterial and venous anastomoses, use of additional venous drainage; (iii) Outcomes: length of hospital stay, recipient site complications (arterial insufficiency, venous insufficiency, flap failure), donor site complications (seroma, wound dehiscence), need for re-exploration.

Complications were defined using standardised criteria. Seroma was defined as a clinically detectable fluid collection requiring aspiration or drainage. Wound dehiscence was defined as the separation of wound edges requiring intervention. Flap failure was defined as complete flap loss requiring removal. Follow-up duration was calculated from the surgery date to the last clinical encounter.

Statistical methods

Continuous variables are reported as means with ranges. Categorical variables are reported as frequencies and percentages. Given the small sample size, formal hypothesis testing has limited power. Fisher's exact test was used for the comparison of categorical variables between groups. A p-value <0.05 was considered statistically significant. No adjustments were made for multiple comparisons. Statistical analysis was performed using Jamovi 2.7.9.0 (https://www.jamovi.org/).

Sample size and power

No formal sample size calculation was performed as this was a retrospective study with a small cohort. Given the small SIEA cohort (n=5), the study has limited statistical power to detect differences in complication rates. Therefore, this study should be considered descriptive and hypothesis-generating rather than definitive.

## Results

Patient characteristics

During the study period from January 2020 to September 2025, 161 patients underwent abdominal-based free flap breast reconstruction with a total of 170 flaps performed. Five patients (3.1%) received SIEA flaps (five flaps in total, all unilateral), while 156 patients (96.9%) received DIEP flaps (165 flaps in total).

Patient demographics are summarised in Table 1. The mean age was 48.4 years (range, 35-67 years) in the SIEA cohort versus 53.5 years (range, 37-74 years) in the DIEP cohort. One SIEA patient (20%) was a current or former smoker compared to 10 DIEP patients (6.4%). None of the patients in either cohort had diabetes mellitus. BMI distribution differed between groups: in the SIEA cohort, three patients (60%) had underweight/normal BMI (18.5-24.9), one patient (20%) was overweight (25-29.9), and one patient (20%) was obese (≥30). In the DIEP cohort, 24 patients (15.4%) were underweight (<18.5), 89 patients (57.1%) had normal BMI, 44 patients (28.2%) were overweight, and no patients were obese. All SIEA reconstructions were performed as immediate reconstructions. In the DIEP cohort, 148 flaps (89.7%) were immediate reconstructions, six flaps (3.6%) were delayed reconstructions, and six patients underwent bilateral reconstruction.

**Table 1:** Patient demographics and clinical characteristics

Characteristic	SIEA (n=5 patients)	DIEP (n=156 patients)
Age (years), mean (range)	48.4 (35-67)	53.5 (37-74)
Current or former smoker, n (%)	1 (20.0)	10 (6.4)
Diabetes Mellitus, n (%)	0 (0)	0 (0)
BMI Category, n (%)		
Underweight (<18.5)	0 (0)	0 (0)
Normal (18.5-24.9)	3 (60.0)	24 (15.4)
Overweight (25-29.9)	1 (20.0)	89 (57.1)
Obese (≥30)	1 (20.0)	44 (28.2)
Flap Configuration, n (%)		
Total flaps performed	5	165
Unilateral reconstruction	5 (100)	159 (96.3)
Bilateral reconstruction	0 (0)	6 (3.6)
Reconstruction Timing, n (%)		
Immediate	5 (100)	148 (89.7)
Delayed	0 (0)	17 (10.3)
Adjuvant Therapy, n (%)		
Preoperative radiation	1 (20.0)	16 (10.3)
Preoperative chemotherapy	2 (40.0)	41 (26.3)
Recipient Vessels, n (%)		
Internal mammary artery/vein	5 (100)	165 (100)
Thoracodorsal vessels	0 (0)	0 (0)
Additional Venous Drainage, n (%)	0 (0)	22 (13.3)

Prior to reconstruction, one SIEA patient (20%) had received radiation therapy compared to 16 DIEP patients (10.3%). Two SIEA patients (40%) had received preoperative chemotherapy compared to 41 DIEP patients (26.3%).

Operative details

All five SIEA flaps were anastomosed to the internal mammary artery (IMA) at the 4th intercostal space to optimise vessel size matching. In the DIEP cohort. All 156 flaps were anastomosed to the IMA. In the DIEP cohort, 22 flaps (14.1%) utilised dual venous drainage. Complete operative details, including operative time, ischaemia time, and blood loss, were not consistently documented in the retrospective records.

Complications

Complication rates are summarised in Table 2.

**Table 2:** Postoperative complications P-values calculated using Fisher's exact test for categorical variables. Statistical power is limited by small SIEA sample size.

Complication(s)	SIEA (n=5 flaps), n (%)	DIEP (n=165 flaps), n (%)	p-value
Recipient Site			
Arterial insufficiency	0 (0%)	0 (0%)	1.00
Venous thrombosis	0 (0%)	1 (0.6%)	1.00
Flap failure	0 (0%)	0 (0%)	1.00
Reexploration	0 (0%)	2 (1.2%)	1.00
Donor Site			
Abdominal seroma	1 (20.0%)	3 (1.8%)	0.058
Abdominal dehiscence	0 (0%)	3 (1.8%)	1.00
Overall			
Any complication	1 (20.0%)	9 (5.5%)	0.264
Hospital Stay			
Mean days	10	8	N/A

Recipient Site Complications

No arterial insufficiency or flap failure occurred in either cohort. No venous insufficiency occurred in the SIEA cohort, while one DIEP flap (0.6%) developed venous congestion (p=1.00). No re-explorations were required in the SIEA cohort compared to two re-explorations (1.2%) in the DIEP cohort (p=1.00).

Donor Site Complications

Abdominal seroma occurred in one SIEA patient (20%) versus three DIEP patients (1.8%) (p=0.058). The single SIEA seroma resolved with conservative management. No abdominal wound dehiscence occurred in the SIEA cohort compared to three cases (1.8%) in the DIEP cohort (p=1.00).

Overall Complications

The overall complication rate was 20% (1/5 patients) in the SIEA cohort versus 5.5% (9/165 flaps) in the DIEP cohort (p=0.264).

Hospital Stay

Mean length of hospital stay was 10 days (range, 8-12 days) for SIEA patients versus eight days (range, 5-14 days) for DIEP patients. The longer stay in the SIEA cohort was attributable to institutional practice patterns during the learning curve, and one patient requiring seroma management.

## Discussion

**Figure 7:**
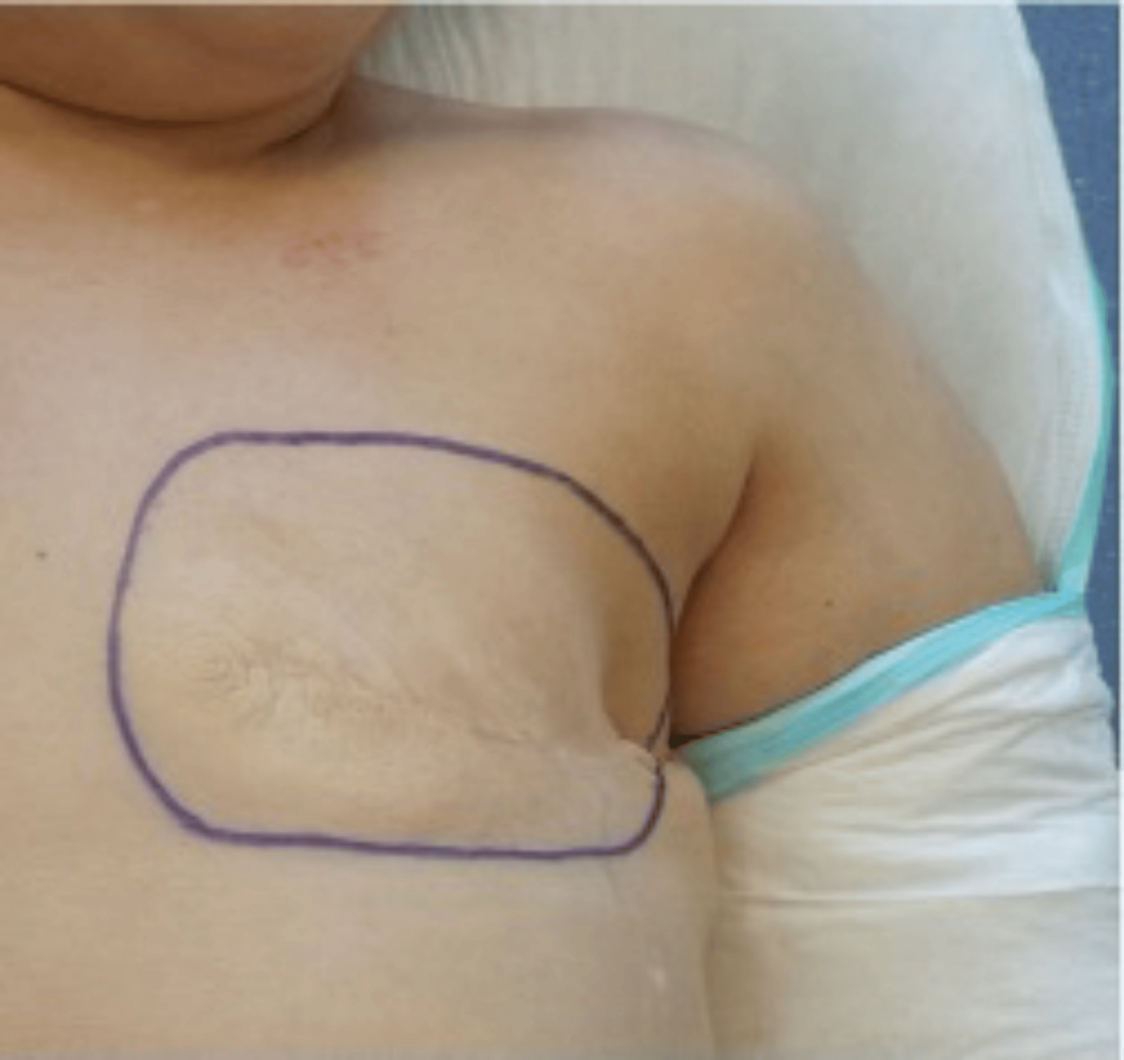
Preoperative left mastectomy defect

**Figure 8:**
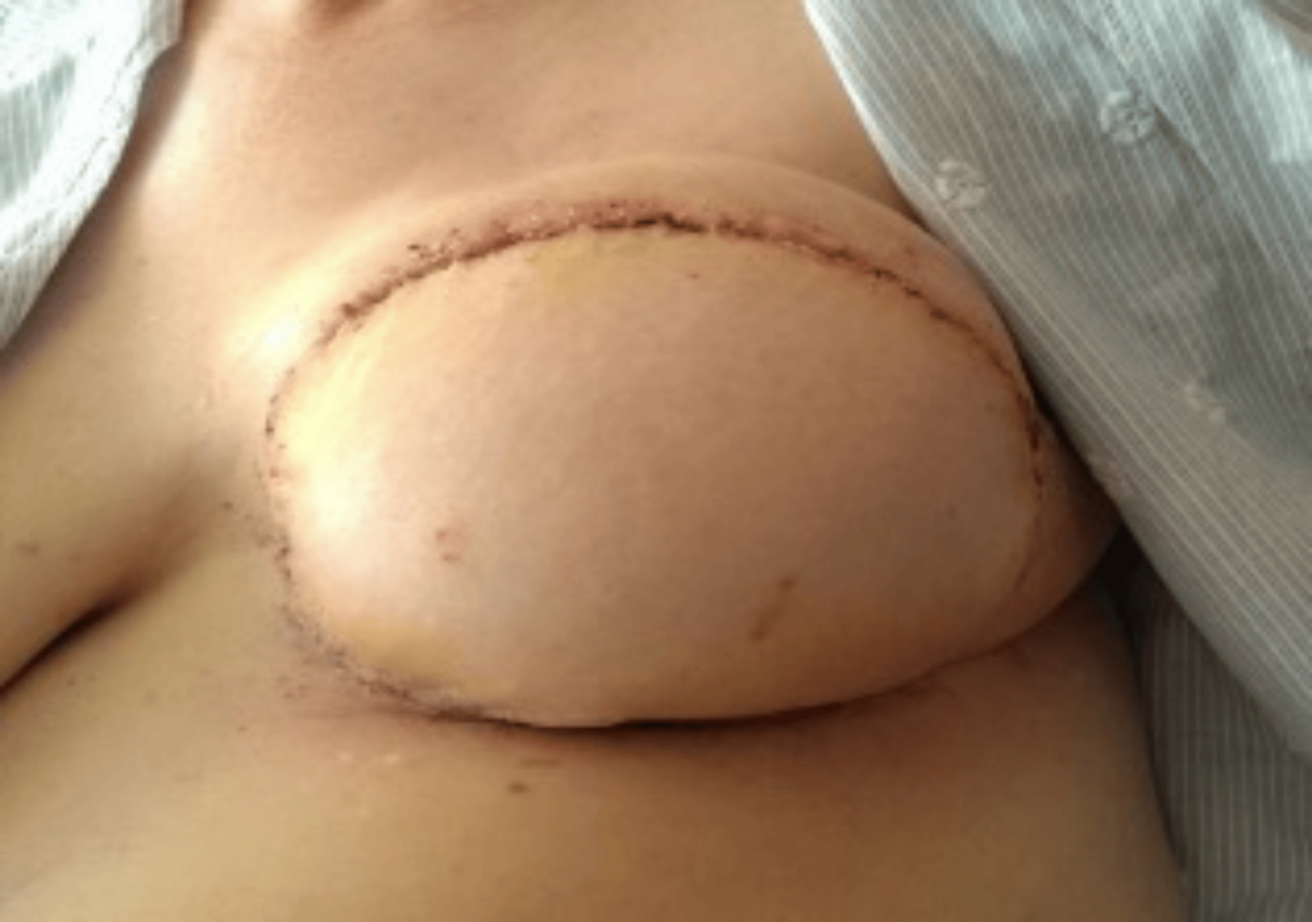
SIEA flap, postoperative day 7 SIEA: superficial inferior epigastric artery

**Figure 9:**
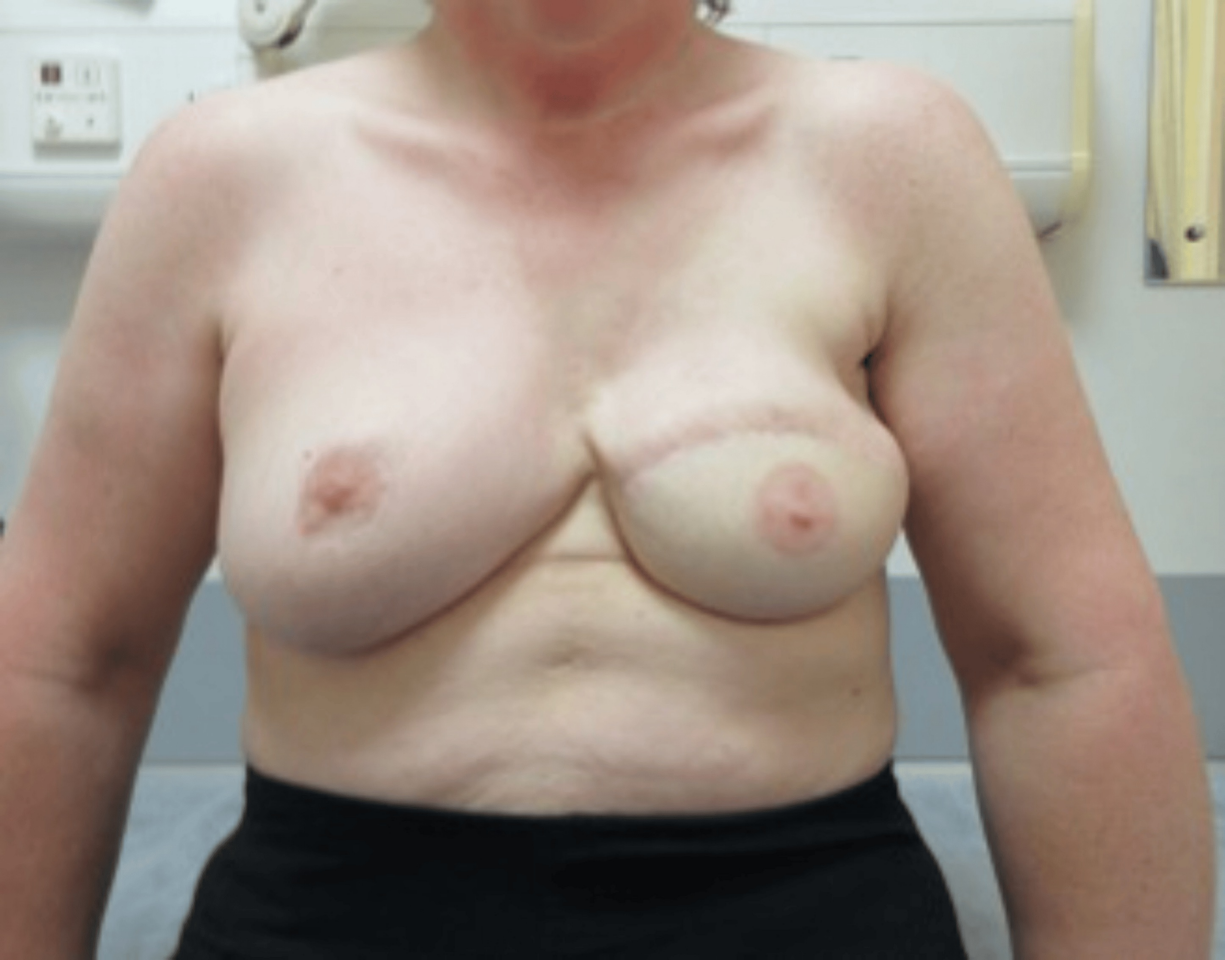
Left-sided SIEA flap with delayed nipple reconstruction and tattooing, five years postoperative SIEA: superficial inferior epigastric artery

This cohort study describes our institutional experience with SIEA flaps for breast reconstruction in five carefully selected patients with favourable superficial arterial anatomy on preoperative CTA. While the small sample size limits definitive conclusions, our results suggest that SIEA flaps can achieve successful outcomes comparable to DIEP flaps when strict selection criteria are applied, and specific technical pearls are implemented (Figures 7-9).

Comparison with published literature

Our findings contrast with earlier reports of high SIEA failure rates. Coroneos et al. reported a 14% total flap loss rate with SIEA flaps, all attributable to arterial thrombosis, compared to 2% with DIEP flaps [10]. Similarly, Selber et al. found a 17.4% vessel thrombosis rate requiring revision with SIEA flaps versus 6.0% with muscle-sparing TRAM flaps, leading them to abandon the technique [13]. In our series, no arterial complications, re-explorations, or flap failures occurred, which we attribute to strict patient selection based on vessel diameter criteria (≥1.5 mm) and recipient vessel matching at the 4th intercostal IMA.

Our approach aligns with more recent studies emphasising refined selection criteria. Spiegel and Khan reported that implementing an intraoperative algorithm requiring SIEA diameter ≥1.5 mm at the lower abdominal incision eliminated flap failures after their initial learning curve [18]. Henry et al. similarly demonstrated that SIEA diameter ≥2.0 mm at its origin on preoperative CTA provides a reliable criterion for successful flap utilisation [17]. Data from Somers et al.'s study showed comparable thrombosis rates (4.5% for superficial flaps vs 0.7% for DIEP, p=0.015) with no significant difference in flap failure rates when using similar selection criteria [20].

Donor site seroma

The trend toward increased seroma formation in our SIEA cohort (20% vs 1.8%, p=0.058) aligns with published literature. Erdmann-Sager et al. reported a 30.7% seroma rate with SIEA flaps compared to 7.0% with DIEP flaps in a large multi-center study [16]. Sadeghi and Malata documented that SIEA flaps require more extensive subcutaneous groin dissection with greater disruption of lymphatic channels, predisposing to seroma formation [14]. Several risk factors contribute to this complication, including bilateral reconstruction, obesity, and the inherent need for suprafascial dissection in SIEA flaps [14,15].

Importantly, the single seroma in our series was managed conservatively. This contrasts with the persistent seromas requiring surgical intervention reported in some series [14]. Our approach of preserving lymphatic tissue around the pedicle, multilayer closure with progressive tension sutures, and prolonged drain placement may have contributed to the relatively favorable outcome.

Technical pearls and innovations

Our experience has identified several technical considerations critical to SIEA success (Table 3).

**Table 3:** Technical challenges and surgical pearls for SIEA flap breast reconstruction IMA: internal mammary artery; IMV: internal mammary vein; SIEV: superficial inferior epigastric vein; SIEA: superficial inferior epigastric artery; CTA: computed tomography angiography; DIEP: deep inferior epigastric artery perforator References: [4-7]

Challenge	Technical Pearl	Rationale
Vessel Size Mismatch	Use 4th intercostal space for IMA anastomosis.	IMA caliber at 4th rib space (1.5-2.5 mm) closely matches SIEA diameter (1-2 mm). IMV bifurcates at 4th space, enabling dual venous anastomosis.
Small Venous Caliber	Perform dual venous drainage (SIEV + venae comitantes) when SIEV <2.5 mm.	Augments venous outflow and provides safety margin against venous congestion.
Risk of Arterial Thrombosis	Strict vessel selection: SIEA diameter ≥1.5 mm at lower abdominal incision. Delicate vessel handling. Avoid pedicle kinking during inset.	Smaller SIEA diameter in comparison to the IMA causes turbulent flow that leads to clot formation. Axial pedicle entry predisposes to kinking can impede blood flow and cause thrombosis.
Flap Perfusion Uncertainty	Intra-operative perfusion assessment with indocyanine green angiography. Temporarily clamp DIEP perforators and assess flap perfusion before committing to SIEA. Design flap wider laterally to include multiple SIEA branches.	Confirms adequate perfusion across midline zones before pedicle division. Additional vascular territories enhance medial perfusion.
Short Pedicle Length	Create generous subcutaneous tunnel. Meticulous flap positioning to avoid tension or compression.	Short pedicle length is inherent limitation of SIEA anatomy. Careful inset technique prevents mechanical complications.
Donor Site Seroma Risk	Preserve lymphatic tissue around pedicle. Minimise cautery in groin. Multilayer closure with progressive tension sutures. Maintain drains until output <30 mL/day × 2 days.	Extensive subcutaneous dissection disrupts lymphatics. Quilting sutures obliterate dead space. Prolonged drainage allows lymphatic sealing.
Patient Selection Uncertainty	Preoperative CTA with assessment of SIEA diameter, length, and course, SIEV diameter and drainage pattern, and adequacy of DIEP system.	CTA allows objective assessment of vascular anatomy and appropriate flap selection.

Limitations of the study

This study has several important limitations that must be acknowledged. The severely limited SIEA cohort (five flaps in five patients) represents the most significant limitation. This small sample provides insufficient statistical power to detect meaningful differences in complication rates. For example, to detect a 15% absolute difference in complications with 80% power would require approximately 140 patients per group. Therefore, our findings should be considered descriptive and hypothesis-generating rather than definitive. Secondly, as a retrospective chart review, this study is subject to information bias (incomplete data capture), recall bias, and the inability to control confounding variables. Operative details such as operative time, ischemia time, and blood loss were not consistently documented, limiting comprehensive comparison. Finally, patients selected for SIEA reconstruction had specific, favorable anatomic criteria, while DIEP patients represented a broader population. This selection bias limits the generalisability of our findings and may contribute to favorable SIEA outcomes through patient selection rather than technique superiority.

## Conclusions

In patients with favourable superficial arterial anatomy on preoperative CTA, SIEA flaps can achieve successful breast reconstruction with minimal donor site morbidity and complete preservation of abdominal wall integrity. While limited by a small sample size, our case series demonstrated no arterial complications or flap failures when strict vessel diameter criteria (≥1.5 mm) were applied, and technical pearls, including recipient vessel matching at the 4th intercostal IMA and dual venous drainage, were implemented. The trend toward increased seroma formation warrants attention but did not require surgical intervention in our experience.

SIEA flaps should not be considered a routine alternative to DIEP reconstruction, but rather a valuable option for the subset of patients with dominant superficial vasculature and inadequate deep perforator anatomy on preoperative imaging. Careful patient selection, meticulous surgical technique, and appropriate expectations are essential to optimise outcomes. Larger multi-institutional prospective studies are needed to definitively establish the safety and efficacy of SIEA flaps compared to DIEP reconstruction and to refine patient selection criteria.

## References

[1] Grotting JC (1991). The free abdominoplasty flap for immediate breast reconstruction. Ann Plast Surg.

[2] Koshima I, Soeda S (1989). Inferior epigastric artery skin flaps without rectus abdominis muscle. Br J Plast Surg.

[3] Allen RJ, Treece P (1994). Deep inferior epigastric perforator flap for breast reconstruction. Ann Plast Surg.

[4] Keller A (2001). The deep inferior epigastric perforator free flap for breast reconstruction. Ann Plast Surg.

[5] Blondeel PN, Beyens G, Verhaeghe R (1998). Doppler flowmetry in the planning of perforator flaps. Br J Plast Surg.

[6] Arnez ZM, Khan U, Pogorelec D, Planinsek F (1999). Rational selection of flaps from the abdomen in breast reconstruction to reduce donor site morbidity. Br J Plast Surg.

[7] Guerra AB, Metzinger SE, Bidros RS (2004). Bilateral breast reconstruction with the deep inferior epigastric perforator (DIEP) flap: an experience with 280 flaps. Ann Plast Surg.

[8] Rozen WM, Ashton MW, Pan WR, Taylor GI (2007). Raising perforator flaps for breast reconstruction: the intramuscular anatomy of the deep inferior epigastric artery. Plast Reconstr Surg.

[9] Menn ZK, Spiegel AJ (2013). Selection of an appropriate method of breast reconstruction: factors involved in customizing breast restoration. Breast Reconstruction - Current Perspectives and State of the Art Techniques.

[10] Coroneos CJ, Heller AM, Voineskos SH, Avram R (2015). SIEA versus DIEP arterial complications: a cohort study. Plast Reconstr Surg.

[11] Vanschoonbeek A, Fabre G, Nanhekhan L, Vandevoort M (2016). Outcome after urgent microvascular revision of free DIEP, SIEA and SGAP flaps for autologous breast reconstruction. J Plast Reconstr Aesthet Surg.

[12] Wolfram D, Schoeller T, Hussl H, Wechselberger G (2006). The superficial inferior epigastric artery (SIEA) flap: indications for breast reconstruction. Ann Plast Surg.

[13] Selber JC, Samra F, Bristol M, Sonnad SS, Vega S, Wu L, Serletti JM (2008). A head-to-head comparison between the muscle-sparing free TRAM and the SIEA flaps: is the rate of flap loss worth the gain in abdominal wall function?. Plast Reconstr Surg.

[14] Sadeghi A, Malata C (2013). Persistent seromas in abdominal free flap breast reconstruction: case reports and literature review. Eplasty.

[15] Merchant A, Speck NE, Michalak M, Schaefer DJ, Farhadi J (2022). Comparing seroma formation at the deep inferior epigastric perforator, transverse musculocutaneous gracilis, and superior gluteal artery perforator flap donor sites after microsurgical breast reconstruction. Arch Plast Surg.

[16] Erdmann-Sager J, Wilkins EG, Pusic AL (2018). Complications and patient-reported outcomes after abdominally based autologous breast reconstruction: results from the multicenter TOPS study. Plast Reconstr Surg.

[17] Henry FP, Butler DP, Wood SH, Jallali N (2017). Predicting and planning for SIEA flap utilisation in breast reconstruction: an algorithm combining pre-operative computed tomography analysis and intra-operative angiosome assessment. J Plast Reconstr Aesthet Surg.

[18] Spiegel AJ, Khan FN (2007). An Intraoperative algorithm for use of the SIEA flap for breast reconstruction. Plast Reconstr Surg.

[19] von Elm E, Altman DG, Egger M, Pocock SJ, Gøtzsche PC, Vandenbroucke JP (2008). The Strengthening the Reporting of Observational Studies in Epidemiology (STROBE) statement: guidelines for reporting observational studies. J Clin Epidemiol.

[20] Somers S, Foley B, Dadzie A (2026). A comparison of SIEA/SCIA and DIEP flaps for autologous breast reconstruction. J Reconstr Microsurg.

